# Supplementary material for: Beyond financial conflicts of interest: Institutional oversight of faculty consulting agreements at schools of medicine and public health
Source: PLoS One. 2018 Oct 29;13(10):e0203179. doi: 10.1371/journal.pone.0203179 (PMC6205599; doi:10.1371/journal.pone.0203179)
Supplement: S1 Appendix — (PDF) [file pone.0203179.s001.pdf]

S1 APPENDIX

Supplemental Material for

Mello MM et al., “Beyond Financial Conflicts of Interest: Institutional Oversight of Faculty Consulting Agreements at Schools of Medicine and Public Health”

1. Process for Calculating Response Rate

Figure S1 provides additional details about the construction of our sample of key informants.

Figure S1. Process for Calculating Denominator for Response Rate

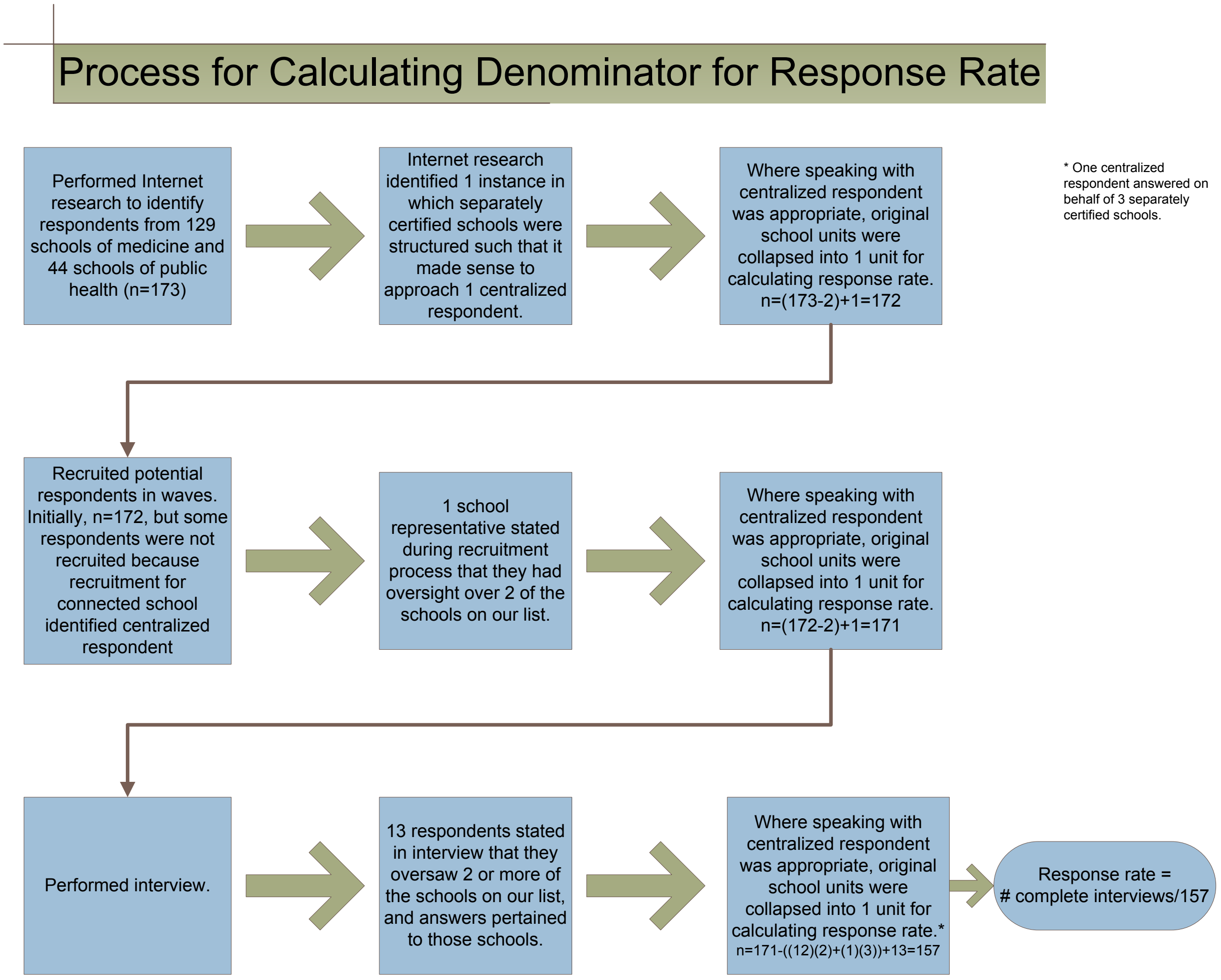

2. Interview Guide

The full text of the interview guide is provided below.

Administrator Interview Guide

Thanks very much for agreeing to talk with me today. Our interview should take about 20 minutes. I am not taping our conversation, but I will be taking notes. If you prefer not to answer a particular question, or don't know the answer, just let me know.

As a reminder, this study is about consulting relationships that your faculty members have with companies. By "consulting relationship," I mean a relationship through which a faculty member receives payment for providing advice or services to companies whose products or services are related to the faculty member's area of scientific expertise. By "consulting relationship" I do NOT mean relationships though which faculty members engage in sponsored research in a form of a grant or contract to their institution, receive speakers' fees, or are considered a regular employee of the company.

I am going to ask you some questions about consulting agreements, by which I mean written agreements for a faculty member to provide advice or services to a company related to his or her area of scientific expertise.

Do you have any questions before we get started?

1. Does your institution routinely review faculty consulting agreements? [If respondent indicates only some kind of consulting agreements are reviewed, probe for which kinds.]

IF NO, SKIP TO QUESTION 9.

2. Is review mandatory or optional?
    a. If mandatory: Why did your institution choose to make institutional review of consulting agreements mandatory?
    b. If optional:
        i. Why did your institution choose to make institutional review of consulting agreements optional rather than mandatory?
        ii. How do you think your faculty would react if the school started requiring review of consulting agreements?

3. Can you describe the review process to me?
    a. Who reviews agreements? What qualifications do they have? What office are they part of?
    b. At what point in the process of executing consulting agreements does the review occur?
    c. What do reviewers look for?
    d. Do reviewers utilize any written review tools, such as guidelines or a checklist?
    e. What do reviewers do if they are concerned about provisions in a draft consulting agreement? [Probe for what role, if any, office plays in negotiating the terms of consulting agreements; how often changes tend to get made; what power institution has to insist on changes]

    f. How long does it typically take to get consulting agreements reviewed and signed?

4. Can you recall any provisions in either initial or final versions of consulting agreements that concerned you because they might restrict a faculty member's academic freedom?
    a. Can you give me some examples of such provisions?
    b. How commonly do you encounter such provisions?

Although we have been talking about consulting agreements with for-profit companies, can you recall other organizations, such as government agencies or nonprofit organizations, that have proposed provisions in consulting agreements that concerned you because they might restrict a faculty member's academic freedom?

    a. Can you give me some examples of such organizations?

5. About how many consulting agreements does your office review in a year?

6. Do you keep copies of the consulting agreements you review?

7. Do you retain any other information about consulting agreements? If so, what?

8. Would you be willing to share copies of a few consulting agreements, after deleting information identifying individuals, your institution, and companies, as well as dollar amounts?
    a. Would other information need to be deleted before you could share the agreements with us?
    b. If yes, will you send us the initial and final versions of the most recent agreement in your possession, with deletions as needed? Is there someone else we should contact about receiving the agreement?

SKIP TO QUESTION 13.

9. Why is institutional review of faculty consulting agreements not conducted?

10. Has your institution considered offering or requiring review of faculty consulting agreement?

11. How do you think your faculty would react if the school started **offering** review of consulting agreements on an optional basis?

12. How do you think your faculty would react if the school started **requiring** review of consulting agreements?

13. Aside from reviewing consulting agreements, what, if any, other types of institutional oversight of faculty consulting relationships does your institution utilize?

14. Does your institution have written policies concerning faculty consulting relationships that speak specifically to consulting agreements (for example, policies about provisions that are and are not acceptable in consulting agreements)?
    a. If yes:
        i. What are the policies focused on?
        ii. Why were the policies adopted?
        iii. Would you be willing to share these policies with us?

15. In what ways, if any, do you perceive faculty consulting relationships affect the interests of their institution?

Finally, I just have a couple of questions about your institution's characteristics.

16. For which institutions does your office handle consulting agreements or other issues related to research administration? (Single hospital, Multi-hospital system, medical school, school of public health, entire university, other?)

17. Thinking about the dollar value of all research funding for these institutions over the last 3 years, about what percentage came from each of the following types of sponsors?
    Industry sponsors?
    Government sponsors?
    Foundation & other external sponsors
    Internal sponsors?
