# Supplementary material for: Beyond financial conflicts of interest: Institutional oversight of faculty consulting agreements at schools of medicine and public health
Source: PLoS One. 2018 Oct 29;13(10):e0203179. doi: 10.1371/journal.pone.0203179 (PMC6205599; doi:10.1371/journal.pone.0203179)
Supplement: S1 Data Coding Instrument — (PDF) [file pone.0203179.s002.pdf]

Background Information*

This instrument shows how the investigators coded the interview notes into structured data. It can be readily crosswalked with the dataset provided to understand what each variable represents. (The dataset omits fields containing potentially identifying information, e.g. NIH rank).

Instructions for Coding:

1. Using "Coding Study IDs" spreadsheet on S: drive, identify the Coding ID for interview. Either create entry with Coding ID, or open existing entry.

2. Enter the Interview Study ID in appropriate field.

3. Read through entire notes of the interview.

4. Read through notes for a second time, coding as you read. Follow the instructions for individual sections, and fields. Starred data collection sections should be completed for EVERY interview.

5. If a relevant comment appears for one field in a question that correlates with a different field, coders should be sure to enter information in relevant field.

6. If interview notes include information about another school (for which respondent is not primarily answering), determine that school's Coding Study ID and (1) enter information into that entry; (2) add the Interview Study ID to that entry; (3) update the Number of Individuals Providing Information field; and (4) update the "Coding Study IDs" spreadsheet.

7. Once notes are coded, change each form's status to complete. This MUST be done for each form, even if not relevant to that interview.

Coding ID	10<br>(Refer to "Coding Study IDs" spreadsheet for this field.)
Interview ID #1	10<br>(Enter interview ID. If multiple interviews contain information for school, enter additional Interview IDs in subsequent fields.)
Interview ID #2	______________________________
Interview ID #3	______________________________
Number of Individuals Providing Information	1<br>(Enter the number of respondents contributing information.)

Respondent Characteristics

Type of School Represented

- ☒ School of Medicine or affiliated research institute
- ☐ School of Public Health
- ☐ 2 or more affiliated schools

(Refer to "Coding Study IDs" spreadsheet for this field.)

Does respondent or someone in their immediate office actually review agreements?

- ☐ No
- ☒ Yes
- ☐ Unclear from interview notes

(Question was not asked. Determine from interview, if possible. If multiple respondents, answer "yes" if true for any of respondents.)

NIH funding rank:

____

(Available at: http://www.brimr.org/NIH_Awards/NIH_Awards.htm)

General Comments*

Complete for every school.

This section deals with recurring themes that were not explicitly asked in the interview. Coders should return to this section after coding the rest of the notes to finalize coding.

Process of oversight is in transition	☐ Mentioned by respondent<br>☐ Statement to the contrary<br>☒ Not mentioned by respondent
Describe transition	(Copy excerpt from notes about transition. If relevant notes
Chair, department head or similar figure may review agreements, but respondent doubts they have expertise in contractual review.	☐ Mentioned by respondent<br>☐ Statement to the contrary<br>☒ Not mentioned by respondent
Suspects many faculty members do not comprehend or read their consulting agreements.	☐ Mentioned by respondent<br>☐ Statement to the contrary<br>☒ Not mentioned by respondent
Consulting agreements/arrangements are considered a private matter between faculty members and the company.	☐ Mentioned by respondent<br>☐ Statement to the contrary<br>☒ Not mentioned by respondent
Comments about the history of school's oversight process	☒ Mentioned by respondent<br>☐ Not mentioned by respondent<br>(This field should focus on changes in the approach taken, not simply the history that led to the adoption of the current approach.)
Description of history of oversight process	review mandatory for 6-7 years<br>(Copy excerpts describing past approaches to consulting arrangements/agreements that are not captured directly by questions asked in interview.)
Faculty members complain that existing policies are too restrictive	☐ Mentioned by respondent<br>☐ Statement to the contrary<br>☒ Not mentioned by respondent
Comments expressing doubt that oversight approach is actually adhered to	☐ Mentioned by respondent<br>☐ Statement to the contrary<br>☒ Not mentioned by respondent
Mentions that someone at the school (formally or informally) often recommends a faculty member get outside legal advice	☒ Mentioned by respondent<br>☐ Statement to the contrary<br>☐ Not mentioned by respondent<br>(This will likely appear in other answers as well. Code here even if comment appears in context that can be coded elsewhere.)

Review Status*

Complete for every school.

Does your institution routinely review faculty consulting agreements?(Choose all that apply. Question asked during interview had more limited responses. Code based on entire description in notes)

- ☐ No review
- ☐ No official review, but done as a favor when asked
- ☐ Review is optional, and occurs as part of official duties when FM asks.
- ☐ Prior review is optional but only provided upon certain triggering circumstances, or for certain people
- ☐ Prior review is mandatory only upon certain triggering circumstances, or for certain people
- ☒ Review is mandatory
- ☐ School tries to gain control over consulting arrangements, often becoming a party to the agreement. Agreements are reviewed when activity is brought within university. Other agreements/arrangements may be looked at to see if they can be brought within the university. [If other agreements are actually reviewed, check the appropriate box above.]
- ☒ School requires addendum or provisions be added to agreement, or that substantively very similar terms are included in agreement. [Check if addendum/provisions are almost always included/strongly rec'd, but FMs can work with school to get similar provisions approved.]
- ☐ School provides FMs with optional addendum/provisions. [Check if tools are available but merely as an option. They need not be used regularly.]
- ☐ Review may occur as part of COI annual disclosure process [If answers indicate review is primarily occurring as part of COI management AFTER execution, skip questions re review process.]
- ☐ Other
- ☐ Process is unclear from interview notes

What is the triggering circumstance, or for which people is review available/required? ______________________ (Excerpt from notes.)

If other, describe process. (Excerpt from notes, if possible.)

Reasons institution chose to make institutional review of consulting mandatory:(Check all that apply. Include responses about why review is mandatory for certain people or upon a triggering circumstance.)

- ☐ Out of a sense of it being the responsible thing for the institution to do
- ☐ In response to publicity surrounding conflict of interest issues, or in an effort to avoid potential negative publicity
- ☐ Concern about IP implications of agreements
- ☐ Mistrusted faculty to report consulting income accurately
- ☐ To protect institution's interests
- ☐ To protect faculty members' interests
- ☐ Other
- ☒ Respondent unsure of reason
- ☐ Reason unclear from notes
- ☐ Review is not mandatory

If other, describe: (Excerpt from notes, if possible.)

Reasons institution chose to make institutional review of consulting agreements optional rather than mandatory(Check all that apply. Include responses about why review is optional for certain people, or in certain situations. Review as a courtesy is not optional.)

- ☐ Agreements are faculty members' private agreements, outside of professional obligations.
- ☐ Mandatory review requires too many resources
- ☐ Review is offered mainly as a service to faculty members
- ☐ Other
- ☐ Respondent unsure of reason
- ☐ Reason unclear from notes
- ☒ Review is not optional

If other, describe: (Excerpt from notes, if possible.)

Reactions to Hypothetical Rev

Complete for every school.

How do you think your faculty would react if the school started offering review of consulting agreements on an optional basis? CHARACTERIZE GENERAL RESPONSE HERE

- ☐ Mainly positively
- ☐ Reluctantly, but most would accept it
- ☐ Mainly indifferently
- ☐ Mainly negatively
- ☐ Reaction would be mixed; some would welcome it, some would be very upset
- ☐ Unsure
- ☐ Unclear from interview notes

(Some respondents provided specific reactions, other general. Coders should make a determination about the general reaction, if possible, and code for specific reactions in the following field.)

What types of reactions would you expect from your faculty if the school started offering review of consulting agreements on an optional basis?

INCLUDE MORE SPECIFIC REACTIONS HERE(Choose all that apply.)

- ☐ Some would feel that they have the school's blessing to continue
- ☐ Some would like the protection review provides (e.g. - "they're not lawyers. Don't know what they're reading", "don't need to pay for outside counsel")
- ☐ Majority would take advantage of it
- ☐ Minority would take advantage of it
- ☐ Some indifferent
- ☐ Some would feel it is an intrusion into their personal business
- ☐ Uproar/very upset
- ☐ Concern that it's more bureaucracy (slow the process down)
- ☐ Would depend on context in which implementation was attempted (e.g. - after a scandal, through the faculty senate)
- ☐ Other
- ☐ Review is mandatory (i.e. - question should not have been asked)
- ☐ Respondent only provided general response (coded above)
- ☐ Unclear from notes

If other, describe: (Excerpt from notes, if possible.)

How do you think your faculty would react if the school started requiring review of consulting agreements? CHARACTERIZE GENERAL RESPONSE HERE

- ☐ Mainly positively
- ☐ Reluctantly, but most would accept it
- ☐ Mainly indifferently
- ☐ Mainly negatively
- ☐ Reaction would be mixed; some would welcome it, some would be very upset
- ☐ Unsure
- ☐ Unclear from interview notes

(Some respondents provided specific reactions, other general. Coders should make a determination about the general reaction, if possible, and code for specific reactions in the following field.)

What types of reactions would you expect from your faculty if the school started requiring review of consulting agreements?

INCLUDE MORE SPECIFIC REACTIONS HERE(Choose all that apply.)

- ☐ Some would feel that they have the school's blessing to continue
- ☐ Some would like the protection review provides (e.g. - "they're not lawyers. Don't know what they're reading", "don't need to pay for outside counsel")
- ☐ Majority would comply
- ☐ Minority would comply
- ☐ Some indifferent
- ☐ Some would feel it is an intrusion into their personal business
- ☐ Uproar/very upset
- ☐ Concern that it's more bureaucracy (slow the process down)
- ☐ Would depend on context in which implementation was attempted (e.g. - after a scandal, through the faculty senate, after an optional period)
- ☐ Other
- ☐ Review is mandatory (i.e. - question should not have been asked)
- ☐ Respondent only provided general response (coded above)
- ☐ Unclear from notes

If other, describe: (Excerpt from notes, if possible.)

Review Process

Coders: Interview included a general question about the review process that has been omitted from the coding scheme. Code information provided in response to that question in the provided fields.

Who reviews agreements?

INSTRUCTION TO CODER: If respondent reports review is done informally and not as part of official duties, check that box AND the box indicating where the individual is located.(Choose all that apply.)

- ☐ Department head
- ☐ President/dean/designee
- ☐ Legal counsel
- ☐ Representative from IP office, tech transfer office, or equivalent
- ☐ Representative from conflicts of interest office
- ☒ Representative from research compliance office
- ☐ Representative from research administration office [If repr. is from identified sub-office within the general research admin office, do not select this option.]
- ☐ Representative from contracts office
- ☐ Individual with subject matter expertise (other than dept head)
- ☐ Review done informally, not as part of official duties
- ☐ Other
- ☐ Review is not performed
- ☐ Unclear from notes

If other, describe. (Excerpt from notes, if possible.)

What qualifications do reviewers have?(Choose all that apply. If multiple individuals review, it is OK to select choices that seem in opposition to each other.)

- ☐ Legal training required
- ☒ Qualified by virtue of position, but currently has legal training
- ☐ Qualified by virtue of position, and currently does NOT have legal training
- ☐ Non-legal training/experience required
- ☐ Other
- ☐ Unclear from notes

If other, describe. (Excerpt from notes, if possible.)

At what point in the process of executing consulting agreements does the review typically occur?

- ☐ Prior to the first draft
- ☒ Once in draft form
- ☐ After execution
- ☐ Unclear from interview notes

Does review of agreement occur in multiple stages? For example: Multiple offices review agreement

- ☐ Yes
- ☒ No
- ☐ Unclear from notes

(Question was not asked. Determine from interview, if possible. If there was no suggestion that review occurs in stages and process appears relatively clear, select "no.")

What do reviewers look for in review?(Check all that apply. Boxes may be overlapping. For example, if review considers a consulting policy that addresses conflict of committment both boxes should be checked.)

☒ Conflict of commitment (e.g. - effect on time, overlap with university duties)
☒ Potential conflicts of interest
☐ Existence of statement that obligations to school take precedent over obligations to company
☐ Choice of law and other dispute resolution provisions
☒ Statement describing scope of work
☒ Fair market value
☐ Compliance with consulting policies/outside activities policy
☐ Whether faculty member is asked to endorse a product
☐ Use of institution's property
☒ Violation of state or federal (e.g. - NIH) laws/policies
☐ Relationships of proposed activity to work under institution
☐ Liability issues
☐ Publication restrictions
☐ Statements governing IP rights (e.g. - FMs should not promise any university rights to company)
☐ Existence of statement limiting the use of the research institution/school name in consulting activity
☐ Other
☐ Unclear from notes

If other, describe (Excerpt from notes, if possible.)

What is the role of written tools in review process?(Check all that apply. Note: Not all respondents considered addendums as written tools. Look to entire interview.)

☐ No written tools are used in review
☐ Reviewer uses a checklist
☐ Reviewer uses a school policy or set of guidelines as a checklist
☐ School provides written guidelines to faculty members regarding agreements/consulting
☒ School provides written addendum/standard provisions for inclusion in agreements
☐ Other
☐ Unclear from notes

If other, describe (Excerpt from notes, if possible.)

What is the role of written addendums or standard provisions?

☐ School does not provide written addendums or standard provisions
☒ Schools requires inclusion of/almost always includes written addendums or standard provisions, essentially unchanged
☐ Faculty members have the option to include either written addendums/standard provisions, or like terms within contract
☐ Use of written addendums or standard provisions is recommended
☐ Written addendums or standard provisions are available, but not emphasized
☐ Other
☐ Unclear from notes whether school has written addendums or standard provisions
☐ Unclear from notes how written addendums or standard provisions are used, but they exist

If other, describe how written addendums or standard provisions are used. ________________________________ (Excerpt from notes, if possible.)

What do reviewers do if they are concerned about provisions in a draft consulting agreement?(Check all that apply.)

☐ Refer to or consult with school's legal department
☒ Requires company to agree to pre-written addendum/provisions that trumps provisions in consulting agreement
☒ Identifies problematic provisions that MUST be changed to faculty member. Faculty member negotiates with company. [Include cases where school occasionally gets involved, but FM takes lead.]
☐ Negotiates directly with company to reach agreement that school is satisfied with.
☐ Recommends (but doesn't require) changes to faculty member regarding provisions that impact institutional interests
☐ Recommends (but doesn't require) changes to faculty member regarding provisions that impact personal interests
☐ If problematic provisions are not addressed, can prevent faculty from entering agreement
☒ Highlights problematic provisions for FM's.
☐ Advises faculty to seek own legal counsel
☐ Other
☐ Unclear from notes

If other, describe (Excerpt from notes, if possible.)

How long does it typically take to get consulting agreements reviewed and signed?

☐ Under a week
☐ 1-4 weeks
☐ 1-2 months
☐ 2+ months
☐ Timing depends heavily on the company
☐ Unclear from notes

Does review consider issues of concern to faculty members?(Many schools distinguish between provisions that might be problematic for the institution, versus provisions that might be problematic for FM's, but which do not impact the institution. This question is designed to pick up that distinction.)

☐ Review does not consider these issues
☐ Review does not officially consider these issues, but the school provides faculty with general guidance regarding such issues
☐ Review does not officially consider these issues, but reviewer often informally mentions concerns
☐ Review considers these issues, but school DOES NOT REQUIRE if such provisions look problematic
☐ Review considers these issues, and REQUIRES changes to agreement if such provisions look problematic
☐ Other
☐ Review considers these issues. Unclear how they address them
☒ Unclear from notes whether these issues are considered

If other, describe. (Excerpt from notes, if possible.)

Can you recall any provisions in either initial or final versions of consulting agreements that concerned you because they might restrict a faculty member's academic freedom?

☐ No
☒ Yes
☐ Unsure
☐ Not asked/not answered

Examples of provisions that concerned you because they might restrict a faculty member's academic freedom(Choose all that apply. Include examples, even when respondent was unsure that it restricted academic freedom.)

☐ Provisions relating to ownership of FM's work product (IP)
☐ Provisions that spill into FM's work for institution
☐ Provisions relating to publication restrictions
☒ Provisions that bar FM from entering into certain types of future professional activities
☐ Provisions relating to confidentiality of certain information received by the faculty member from the company
☐ Other

How commonly do you encounter provisions relating to ownership of FM's work product?

- ☐ Rarely
- ☐ Sometimes
- ☐ Commonly
- ☐ Always/almost always
- ☐ Unsure
- ☐ Unclear from notes

How commonly do you encounter provisions that spill into FM's work for institution?

- ☐ Rarely
- ☐ Sometimes
- ☐ Commonly
- ☐ Always/almost always
- ☐ Unsure
- ☐ Unclear from notes

How commonly do you encounter provisions relating to publication restrictions?

- ☐ Rarely
- ☐ Sometimes
- ☐ Commonly
- ☐ Always/almost always
- ☐ Unsure
- ☐ Unclear from notes

How commonly do you encounter provisions that bar FM from entering into certain types of future professional activities?

- ☐ Rarely
- ☒ Sometimes
- ☐ Commonly
- ☐ Always/almost always
- ☐ Unsure
- ☐ Unclear from notes

How commonly do you encounter provisions relating to confidentiality of certain information received by the faculty member from the company?

- ☐ Rarely
- ☐ Sometimes
- ☐ Commonly
- ☐ Always/almost always
- ☐ Unsure
- ☐ Unclear from notes

If other, describe

(Excerpt from notes, if possible.)

How commonly do you encounter these other provisions?

- ☐ Rarely
- ☐ Sometimes
- ☐ Commonly
- ☐ Always/almost always
- ☐ Unsure
- ☐ Unclear from notes

How commonly do you encounter any of these problematic provisions?

- ☐ Rarely
- ☒ Sometimes
- ☐ Commonly
- ☐ Always/almost always
- ☐ Unsure
- ☐ Unclear from notes

(If respondent doesn't indicate the frequency of specific types of provisions indicate here their overall characterization of frequency. If the respondent did indicate frequency for particular types of provisions, but also indicated an overall frequency, answer.)

Can you recall other organizations, such as government agencies or non-profit organizations, that have proposed provisions in consulting agreements that concerned you because they might restrict a faculty member's academic freedom?

- ☒ No
- ☐ Yes
- ☐ Unsure
- ☐ Not asked/Not answered

Examples of such organizations: (Include information provided, even if respondent was unsu

About how many consulting agreements does your office review in a year?

On average, 2 per week. Probably less than 100 per year.
(If entering numbers, do not spell numbers out.)

Do you keep copies of the consulting agreements you review?

- ☐ Never
- ☐ Sometimes
- ☒ Always
- ☐ Unsure
- ☐ Unclear from notes

Do you retain any other information about consulting agreements?

- ☒ No other information is retained
- ☐ Emails about review/agreement are saved
- ☐ Email trail might be saved, but not systematically
- ☐ COI disclosures
- ☐ Documentation of approval of consulting activity
- ☐ Other
- ☐ Unclear from notes

(Check all that apply.)

If other, describe

(Excerpt from notes, if possible.)

Would you be willing to share copies of a few consulting agreements with us?

- ☐ No
- ☐ Maybe
- ☒ Yes
- ☐ Does not retain copies of agreements
- ☐ Not answered/not asked

If other information would need to be deleted before agreements can be shared, describe.

____
(Excerpt from notes, if possible.)

No review

Why is institutional review of consulting agreements not conducted?(Check all that apply. If school reviews in only some circumstances, enter reasons (if given) why review doesn't occur more widely.)

- ☐ Agreements are FM's private agreements, outside of professional obligations
- ☐ Mandatory review requires too many resources
- ☐ Agreement is outside the school's purview
- ☐ Prefer to give clear guidance to faculty about what should and should not appear in a consulting agreement
- ☐ Resistance from within school
- ☐ Belief that conflict of interest procedures address issues without requiring review
- ☐ Never been really considered
- ☐ Other
- ☐ Unsure
- ☐ Unclear from notes

If other, describe. (Excerpt from notes, if possible.)

Has your institution considered offering or requiring review of faculty consulting agreements?

- ☐ No
- ☐ Yes, formally
- ☐ Yes, informally
- ☐ Unsure
- ☐ Unclear from notes

(If respondent provides an answer such as "not that I'm aware of," coders should use their judgment to determine whether it appears that respondent would likely be aware of a formal consideration of review. This may include looking at the position s/he holds.)

Alternatives to Review and Policies*

Complete for every school.

Coders should draw from the entirety of the interview for this section. Respondents often described policies, and alternative processes throughout interview.

Aside from reviewing consulting agreements, what, if any, other types of institutional oversight of faculty consulting relationships does your institution utilize?(Check all that apply. Include responses provided to other questions in interview, but relevant here.)

- ☐ Disclosure of consulting activities outside of conflict of interest disclosures.
- ☒ COI disclosures/development of conflict avoidance and management plans
- ☐ Policy governing what is and isn't an outside activity, and what is and is not permissible
- ☒ Addendum/certain provisions that can/must be added to agreements (may or may not be paired with review)
- ☐ Requirement for approval to participate in consulting activity from dean, chair of department or other official [This approval does NOT require approval of agreement. Review of agreement alone should not be counted here.]
- ☒ Conflict of commitment policies that faculty are required to adhere to, including cap on time consulting
- ☐ Cap on amount faculty can earn through consulting
- ☐ Performs audits/ reserves right to see agreements to ensure FMs are in compliance with school policies
- ☐ Stark/Anti-Kickback Statutes analysis
- ☐ Institution, where possible, transforms consulting arrangement into arrangement where money goes through school (may or may not include review)
- ☐ Training for faculty
- ☐ No other oversight is utilized
- ☐ Other
- ☐ Unclear from notes

If other, describe. (Excerpt from notes, if possible.)

Does your institution have written policies concerning faculty consulting relationships that speak specifically to consulting agreements? For example, policies about provisions that are and are not acceptable in consulting agreements.

- ☐ No
- ☒ Yes
- ☐ Has relevant policies, but unsure if they directly address consulting agreements
- ☐ Unsure
- ☐ Unclear from notes

What are policies focused on?(Choose all that apply. One policy may cover multiple options. Coder shoud look for relevant information in entirity of notes.)

- ☐ Requires that actual provisions/addendum be included in agreements
- ☒ Requires review of consulting agreements
- ☒ Conflict of commitment/outside professional activities
- ☐ Conflicts of interest
- ☐ Intellectual property
- ☐ Consulting, specifically
- ☐ Authorship requirements
- ☐ Publication rights
- ☐ Professional integrity
- ☐ Highlights potentially problematic provisions that FMs should be aware of
- ☐ Requires that scope of work and/or payment of fair market value be defined in written agreement
- ☐ Stark/Anti-Kickback laws
- ☐ Other
- ☐ Unclear from notes

If other, describe. (Excerpt from notes, if possible.)

Why were the policies adopted?(Check all that apply.)

- ☐ No relevant policies
- ☐ In reaction to a requirement from larger health system/university
- ☐ To help clarify questions from faculty
- ☐ To allow patients to better look at potential conflicts their providers might have
- ☒ Due to publicity around these issues (e.g. - Stanford case, Grassley's focus on COI)
- ☐ In response to state/federal laws
- ☐ In response to a negative incident at the institution
- ☐ Other
- ☐ Unsure
- ☐ Unclear from notes

If other, describe. (Excerpt from notes, if possible.)

Would you be willing to share these policies with us?

- ☐ No
- ☒ Yes
- ☐ Maybe
- ☐ Unclear from notes

Impact on School*

Complete for every school.

In what ways, if any, do you perceive that faculty consulting relationships affect the interests of an institution?(Check all that apply.)

- ☐ Can help disseminate knowledge held at the institution
- ☐ Can raise profile of individual
- ☐ Can raise profile of institution
- ☐ Can give faculty real-world experience and allows them to understand what is going on in the field
- ☒ Can build relationships with parties outside the university
- ☒ Allows faculty to supplement their income
- ☐ Can create education, research, and funding opportunities
- ☒ Can exert influence on how faculty members carry out duties at school (COI)
- ☐ Can create conflicts of commitments
- ☐ Can damage reputation of institution (include comments about publicity surrounding poorly managed consulting)
- ☐ Can threaten the integrity of the institution, teaching, or research, generally.
- ☐ Can raise Stark/anti-kickback issues
- ☐ Can pose a threat to school's intellectual property rights
- ☐ No impact
- ☐ Other
- ☐ Unclear from notes

If other, describe. (Excerpt from notes, if possible.)

Additional Comments

Use this section for information that is not captured in any of the other fields.

Additional Comments	Addendum refers to policies that may conflict with the terms of the agreements (IP, publication rights, etc.). These policies trump anything conflicting in the agreement.
